# Theories of Creativity in Music: Students' Theory Appraisal and Argumentation

**DOI:** 10.3389/fpsyg.2021.612739

**Published:** 2021-03-25

**Authors:** Erkki Huovinen

**Affiliations:** Department of Music Education, Royal College of Music in Stockholm, Stockholm, Sweden

**Keywords:** argumentation, creativity, implicit theories, improvisation, lay theories, musical creativity, musical thought, theory choice

## Abstract

Most research on people's conceptions regarding creativity has concerned informal beliefs instead of more complex belief systems represented in scholarly theories of creativity. The relevance of general theories of creativity to the creative domain of music may also be unclear because of the mixed responses these theories have received from music researchers. The aim of the present study was to gain a better comparative understanding of theories of creativity as accounts of musical creativity by allowing students to assess them from a musical perspective. In the study, higher-education music students rated 10 well-known theories of creativity as accounts of four musical target activities—composition, improvisation, performance, and ideation—and argued for the “best theoretical perspectives” in written essays. The results showed that students' theory appraisals were significantly affected by the target activities, but also by the participants' prior musical experiences. Students' argumentative strategies also differed between theories, especially regarding justifications by personal experiences and values. Moreover, theories were most typically problematized when discussing improvisation. The students most often chose to defend the Four-Stage Model, Divergent Thinking, and Systems Theory, while theories emphasizing strategic choices or Darwinian selection mechanisms were rarely found appealing. Overall, students tended toward moderate theory eclecticism, and their theory appraisals were seen to be pragmatic and example-based, instead of aiming for such virtues as broad scope or consistency. The theories were often used as definitions for identifying some phenomena of interest rather than for making stronger explanatory claims about such phenomena. Students' theory appraisals point to some challenges for creativity research, especially regarding the problems of accounting for improvisation, and concerning the significance of theories that find no support in these musically well-informed adults' reasoning.

## Introduction

### Theories and Informal Conceptions Regarding Creativity

General theories of creativity are based on the assumption that there is something we can call human creativity—that we can see creativity as one phenomenon, despite its apparent plurality. Definitions of creativity most typically share such characteristics as uniqueness (or novelty) and usefulness (see Plucker et al., 2004). While often sharing such basic assumptions, most contemporary theories of creativity are rather self-consciously demarcated to addressing only particular aspects of the multifarious phenomenon. This is easy to see in any of the introductory volumes and reviews available on the topic. Runco (2007), for instance, includes separate chapters on cognitive, developmental, biological, clinical, social, educational, historical, cultural, personality-based, and enhancement-oriented theories of creativity. In the present article, I will be referring to Kozbelt et al.'s (2010) review that similarly presents 10 (slightly different) classes of theories (see Appendix 1). Hence, while early theories of creativity might have appeared as unduly focused on cognitive aspects such as Divergent Thinking (Guilford, 1968) or “dissociation” (Koestler, 1964), the contemporary theoretical landscape is broader, addressing questions regarding creative lives, creative collaborations, creative products, the social and societal contexts of creative work, the neurological underpinnings of creativity, and more. It thus also seems clear that different creativity theories may address somewhat different sets of core questions (for a review, see Kaufman and Glăveanu, 2019). Some theories such as Csikszentmihalyi's (1997) Systems Theory take into account the reception of an idea or a product by a field of experts in a sociocultural context. However, many general theories of creativity tend to take a substantialist approach to creativity in the sense that the phenomenon (even in its societal aspects) is treated extrahistorically, as a human attribute, rather than as intertwined in historically contingent discourses and values (see Nelson, 2015, 2018).

Apart from developing scholarly theories of creativity, researchers have also paid attention to practitioners' conceptions and understandings of the phenomenon. This is understandable: any attempts to measure something as multifaceted as creativity could probably benefit from heeding the views of those with experience in the domain in question, in order to judge which aspects are relevant to consider. Artists, in particular, are typically taken as reliable informants about the nature and progress of their own creativity (e.g., Lindauer et al., 1997; Botella et al., 2013; Daniel, 2020), and artists' conceptions of creativity may indeed be richer than is the case for some other professions (Spiel and von Korff, 1998). By contrast, studies of teachers' conceptions of creativity have often emphasized the “informal,” “implicit,” or “everyday” character of their thinking, pointing out informants' misconceptions about the topic. In a review of empirical studies in this area, Mullet et al. (2016) find a difference between descriptors that K-12 teachers typically associate with creative individuals (imaginative, artistic, intellectual, etc.) and researchers' criteria for creativity (fluency, flexibility, etc.), concluding that, overall, “teachers' conceptions of creativity were limited, vague, or confused” (Mullet et al., 2016, p. 27). Whereas some researchers suggest that internal inconsistencies among teachers' beliefs might hinder their efforts to promote students' creativity (Kampylis et al., 2009), Mullet and colleagues go further, suggesting that the discrepancies between teachers' views and research “reflect teachers' difficulties in recognizing an authentically creative student or experience in the classroom” (Mullet et al., 2016, p. 24). In another review on K-12 teachers' conceptualizations of creativity, Andiliou and Murphy (2010) likewise pay attention to misconceptions, stating that the degree to which teachers' understandings of creativity align with researchers' views “becomes and essential issue with practical significance for teachers who wish to identify, develop, and evaluate creative outcomes” (Andiliou and Murphy, 2010, p. 203). These authors thus implicitly subscribe to what we might call *theory optimism* about creativity. This is the view that empirically supported theories of creativity give the best possible approximation about the central matters of fact regarding creativity and that creative phenomena can best be recognized and indeed furthered on the basis of this knowledge.

Influenced by Sternberg (1985), much of the research along these lines has been carried out using the term “implicit theory.” In one of his experiments, Sternberg let laypersons rate how characteristic various behaviors would be for an ideally intelligent, creative, or wise individual. The top 40 behaviors in each case were then used in one of three sorting tasks in another experiment, where students sorted behaviors into piles reflecting which of them were “likely to be found together” in a person. For intelligence, creativity, and wisdom, the respective sorting tasks thus led to multidimensional scaling solutions concerning the dimensions of each of these constructs (ibid.). Such a scaling, of course, depicts the respondents' implicit theories on a group level, and it does not exclude the possibility that various participants' individual implicit theories might be mutually incompatible in some way. For the present purposes, it is interesting that the sorting task itself required the participants, in essence, to arrange the items in a *structure* that suggests a wider system of beliefs. Such a structural aspect warrants the use of the term “theory” in the sense that scientific theories, too, are structured entities (see Winther, 2015) and typically more complex than single beliefs. Guilford (1968, p. 22), for instance, saw theories as “semantic systems.”

In the research concerning implicit conceptions about creativity, psychometric methods may have biased the results toward reporting particular beliefs instead of such larger structures of thought. For instance, many putative misconceptions about creativity—such as the belief that creativity is synonymous with the arts (e.g., Patston et al., 2018)—might be reported by rating a single questionnaire item. Similarly, methods using free association tend to yield lists of characteristics of creativity that may be condensed in categories signified by simple labels such as “beautiful,” “curious,” and “original” (Lothwesen, 2020). In more comprehensive factor-analytical (e.g., Cropley et al., 2019) or correspondence-analytical settings (e.g., Lothwesen, 2020), such beliefs do reveal a larger structure, but this is achieved by the researchers and describes the participants' thinking on a group level. Hence, these studies do not directly address participants' individual commitments to theories (in the sense of belief systems). In their analysis of studies concerning teachers' beliefs about creativity, Andiliou and Murphy (2010) rightly noted that uses of the term “implicit theory” (in Runco et al., 1993; Chan and Chan, 1999; Runco and Johnson, 2002) had been “narrowed and limited to represent beliefs [rather] than a belief system” (Andiliou and Murphy, 2010, p. 206).

In an attempt to transcend a psychometric approach that focuses on the quantification of isolated beliefs, Pavlović and Maksić (2019) studied university teachers' implicit theories of creativity using a qualitative questionnaire. They found five types of implicit theories and made more detailed observations of the contexts of applying the theories, arguing that the informants held individualistic attitudes regarding the general definition of creativity but moved to activity theories when they focused on manifestations of creativity in students. Likewise, several English interview studies with music teachers have suggested that teachers' views regarding creativity can be substantially shaped by their own teaching experiences (Crow, 2008; Odena and Welch, 2009, 2012; Kokotsaki, 2011, 2012). Such studies suggest that practitioners' views concerning creativity may be crucially influenced by the broader contexts in which they are embedded. In turning to examine conceptions of creativity in the domain of music, we should thus be reminded of the vast cultural differences that may exist in the practices and beliefs surrounding music. As Hill (2012) observes in an ethnomusicological setting, varying cultural beliefs about where music comes from may also fundamentally shape perceptions of what musical creativity is and who has the ability to be creative. Again, this underlines the importance of treating conceptions regarding creativity as parts of larger belief systems.

### Theories of Creativity in Music Research

As one of the remarkably creative domains of human activity, music might seem to provide an interesting test case for general theories of creativity. Yet most research on musical creativity takes place in disciplines that are quite separated from general theories of creativity. This is well exemplified by the field of ethnomusicology—an area in which creative activities such as musical improvisation are recurrently studied. For example, none of the 36 chapters in Bruno Nettl's two important anthologies on musical improvisation (Nettl and Russell, 1998; Solis and Nettl, 2009) explicitly builds on any general theories of creativity, although some individual authors discuss such related areas as expertise research (see Pressing, 1998) and the psychology of “flow” (see Campbell, 2009; Turino, 2009), or briefly mention findings in the research on the development of creativity (Campbell, 2009). Rather than framing the phenomenon of musical improvisation by theories of creativity, the authors rely on the rich theoretical tradition of ethnomusicology itself, or find theoretical support from fields such as sociology, anthropology, linguistics, literary studies, semiotics, musicology, music theory, music education, or philosophy. Similar observations could be made in the recently expanding field of so-called critical improvisation studies that covers but is not limited to addressing musical improvisation. Among the 56 main chapters of *The Oxford Handbook of Critical Improvisation Studies* (Lewis and Piekut, 2016), Dean and Bailes (2016) briefly compare Pressing's (1988) theory of improvisation to the Geneplore model of creativity (Finke et al., 1992) while Young and Blackwell (2016) mention Boden's (1990) notion of transformational creativity. Otherwise, only a handful of authors refer to Csikszentmihalyi's “flow,” give references to creativity studies in footnotes, or mention scholars such as Amabile or Simonton, but without referring to their main theoretical contributions in the study of creativity (as reflected in, say, Kozbelt et al., 2010). Such examples might raise some concern: are general theories of creativity perhaps unknown to improvisation scholars or deemed inappropriate or irrelevant by them?

The disregard for general theories of creativity by researchers of particular forms of musical creativity may seem surprising, but it often has good disciplinary reasons. Culturally oriented scholars, for instance, may see some general theories of creativity as too cognitive in their focus or as inappropriately relying on modernist ideologies of individual “innovation.” Thus, drawing on creativity research in fields such as ethnomusicology or media studies might tend to be delimited to theories with a social bent—such as Csikszentmihalyi's (1997) Systems Theory (McIntyre, 2006, 2008; see, e.g., Borgo, 2007) or Sawyer's (2003) work on group creativity (e.g., Borgo, 2007; Schuiling, 2019). Another related aspect is that many culturally oriented music scholars may feel that they are “fighting the good fight against universalizing theories and culture-blind scholarship” (Slominski, 2020, p. 227). An epistemological commitment like this can be hard to square with the apparent generality of creativity theories. Moreover, such disciplinary self-understandings can also be intertwined with writing styles. For instance, some researchers in musicology like to begin their studies *in medias res*, avoiding generalizing theoretical frameworks—something that is amply demonstrated by many of the introductory sections to articles in the abovementioned volumes by Nettl.

But similar sentiments are common in other disciplinary fields, as well, such as in the psychology of music and related empirical disciplines. This is no place for a comprehensive review of the field in which researchers such as Sawyer (2003), Johnson-Laird (2002), and many others have made important contributions to creativity research. What I want to point out is the uneasiness which other prominent researchers have expressed regarding general theories of creativity. In their introduction to the first modern anthology on musical creativity in this area, Deliège and Richelle urged us to “get rid of *creativity*, and look at *creative acts*” (Deliège and Richelle, 2006, p. 2; emphasis in the original). In another relevant anthology, editors Hargreaves et al. (2012) similarly argue against general theories of creativity, writing: “Since creativity actually exists in so many different forms, activities, and contexts, giving rise to an infinitely variable range of products, any attempt to formulate a unitary description or explanation is doomed to failure” (Hargreaves et al., 2012, p. 4). Interestingly, Hargreaves and colleagues also suggest that “a focus on imagination—on internal mental processes—is more useful than one on creativity because it encompasses a much broader range of concepts and behavior” (Hargreaves et al., 2012, p. 3). In this view, then, creativity as a topic seems too limiting (apparently leaving out forms of imagination such as listening that do not involve some kind of product) but at the same time too general to be addressed in unitary theories.

Various strands of scholarly particularism may nevertheless differ between one another in terms of what to do with the concept of creativity. As seen above, some scholars are suspicious of the whole concept, which easily leads to *theory skepticism* regarding any general theories of creativity—often expressed without detailed scrutiny of such theories. As an extreme position, Frith, in discussing power relations in particular domains of record production, extends this skepticism to the domain-specific notion of “musical creativity.” According to his view, this notion “is more of a hindrance than a help in understanding music-making practice,” and thus “we should cease to use the term altogether” (Frith, 2012, p. 71). Other particularists have taken more positive views, trying to save the notion of creativity by insisting on its inherent plurality. Burnard's (2012a) bottom-up sociological accounts of various “musical creativities” provide a case in point. Such views also open the door to questions regarding how some theories of creativity might have something meaningful to say about music. Indeed, asking such questions on a level closer to the phenomena of interest reflects a non-universalizing tendency among general creativity researchers, as well. In the preface to his introduction to theories of creativity, Runco (2007, p. x) suggests that “the creative process is multifaceted” and complex to the extent that an “eclectic approach is necessary.” According to such *theory eclecticism*, the suitability and usefulness of particular theories would always have to be contextually determined. Hence, even if creativity is conceptualized as a unitary phenomenon or as a “distinct and independent capacity” (Runco, 2007), this complex totality would still need various theoretical tools to be properly accounted for. Finally, still another position—we might call it *theory revisionism*—arises out of the concern that mainstream approaches to theorizing about creativity have simply been too individualistic, too mentalistic, or too product-oriented and that the whole field could be reoriented on this level. Most notably, perhaps, there has been growing interest in distributed, ecological, or 4E approaches to creative cognition in music (Linson and Clarke, 2017; van der Schyff et al., 2018; Schiavio et al., 2020). In the work of Clarke and his associates, for example, the distributed nature of musical creativity has been demonstrated through detailed case studies of micro-social interaction and embodied instrumental engagement (Clarke et al., 2013, 2017).

In some areas of music research, a certain theoretical eclecticism regarding general theories of creativity appears to emerge from the larger research field, although rarely as an explicit position of individual researchers. A systematic review of this topic would require a separate undertaking, but some instructive examples can be provided, say, in Collins (2012) anthology on creative processes in musical composition. Of the 11 chapters in the volume, seven explicitly reference one or more general theories of creativity. Some authors address composition as an individual creative process: Katz (2012), for instance, takes her lead from such theories as Galenson's typological scheme of “experimental innovators” and “conceptual innovators” (or “seekers” and “finders”; see Galenson, 2001, 2006, 2009), and Wallas's (1926) Four-Stage Model of creativity—suggesting that creative processes involve successive stages of preparation, incubation, illumination, and verification [Wallas (1926, p. 97 ff.) also paid attention to an “intimation” stage when illumination was imminent]. Wiggins (2012), in turn, theorizes composition relying on Boden's (1990; 2010) ideas of creativity as the exploration or transformation of conceptual spaces. Brown and Dillon (2012) discuss modes of meaningful engagement with musical composition, drawing on de Bono's (1992) thoughts of creativity as finding alternative perceptions or conceptualizations and on Dennett's (2001) pseudo-Darwinian emphasis of exploitation of accidents. Bailes and Bishop (2012) address various forms of compositional imagery, seeing them to align with Ainsworth-Land's (1982) general stage development model of creativity. Among the more socially informed views, Burnard's (2012b) presentation of real-world composition practices is guided by Amabile's (1996) views regarding the social dimensions of creativity, and Bennett's (2012) analysis of collaborative songwriting is influenced by the Systems Theory of creativity. Other authors rely more on theoretical approaches indigenous to the field of music and/or develop their own theoretical models for musical composition.

Even this small collection of examples suggests that the field of creativity research can easily be sampled for support to a wide range of perspectives into a more or less circumscribed form of musical creativity (here, composition)—without much concern for how other, competing theoretical schemes might have handled the task. In Collins' volume, one finds very little explicit argumentation regarding theory choice: many of the authors write as if they would have already made up their minds about which theoretical framework to stand upon. The clearest exception in the anthology appears in Kozbelt's (2012) account of composers' lifespan creativity trajectories. Kozbelt first pits the expertise acquisition view of creativity (Ericsson, 1999) against the Blind Variation and Selective Retention view that emphasizes serendipity in the creative process (Campbell, 1960; Simonton, 1997, 1999, 2010, 2015), noting that these two theories “make radically different assumptions about the fundamental nature of creativity and quite divergent predictions about how creativity unfolds throughout creators' lives” (Kozbelt, 2012, p. 28). Subsequently, Kozbelt argues that results concerning composers' career landmarks are hard to reconcile with the two abovementioned theories but are better accounted for by using Galenson's typological approach. Pending a systematic review of other similar literature in the field, I venture the suggestion that such comparative argumentation about the relative empirical adequacy of creativity theories is rare within music research. Finally, a complementary question that is typically left open in contexts such as the abovementioned anthology is how the chosen theories would fare in the case of other kinds of creative musical activities. The theoretical eclecticism regarding theories of creativity that arises from the combined efforts of music researchers thus tends to leave both theory choice and the scope of the theories inexplicit.

### Rationale for the Present Study

The importance of studying creativity is often taken for granted by researchers (see Forgeard and Kaufman, 2015), but in the case of music this may be less of a problem than in some other fields. Few might question the idea that music is a creative field of human activity. As the above review suggests, however, the relevance of *theories* of creativity for music is less clear. While the position of theory optimism would imply that general, empirically grounded theories of creativity might be used to correct musical practitioners' views and even enhance their creative potential, theory skepticism would claim the primacy of the actual practices, treating any attempts at theoretical systematization with suspicion. In my view, both of these positions are problematic as applied to music. Theory optimism appears complacent: instead of assuming that music specialists' conceptions can offer valuable insights into creativity (see e.g., Koutsoupidou, 2008), it assumes that researchers should start correcting creative practitioners in their views. Moreover, theory optimism might even seem to suggest that creative practices are best furthered by *convergent*, theoretically systematic thinking about creativity—rubbing against the notion of creativity as divergent thinking. Theory skepticism, in turn, would seem to jump to conclusions: against the fact that at least some musical creators and researchers have found use for general theories of creativity, it simply dismisses such examples without empirical scrutiny.

While it may be granted that much informal thinking on creativity can be reflected in simple questionnaire items, the present research was based on the assumption that people might equally well be able to relate to more complex, scholarly theories regarding the topic. Given that the gist of many theories of creativity is expressible in rather non-technical terms (see Appendix 1) and that many of them have been inspired by creative individuals' own reports, we could indeed expect such theories to be understandable to at least educated practitioners in a field such as music. This is also suggested by how creativity theorists often become sought-after speakers outside of the academia. In such contexts, scholars may tend to promulgate their own theoretical views rather than seeking to subject them to comparative scrutiny. At least to my knowledge, there have not been systematic efforts to ask ordinary people or practitioners in a field about their reactions to broader selections of creativity theories. Therefore, we might not even know whether some such theories would tend to be rejected outright by the relevant practitioners themselves. The current study was thus based on curiosity: assuming that musical practitioners' activities are supposed to be covered by general theories of creativity, what would such practitioners themselves say about these accounts? Of course, we cannot expect theories in behavioral sciences to be automatically felled by lack of acceptance by those whose actions are accounted for. Still, some more knowledge about creative people's appraisal of theories concerning their domain would certainly help us untangle some of the knots in the mixed reception that these theories have generated.

In designing the study, I thus tentatively adopted the position of theory eclecticism—not as a given result, but as methodological guidance. The aim was to study the appraisal of theories of creativity among higher-education music students, by building on the assumption that theories might vary in their suitability in accounting for different musical activities. In allowing the participants to engage with theories of creativity, I also wanted to embrace the positive suggestion inherent in theory optimism—that practitioners could be offered information about creativity research. Finally, in asking the participants to evaluate the suitability of such theories for music, I opened the door to such views as theory skepticism, theory eclecticism, and even theory revisionism as possible result scenarios.

In an empirical study of people's theory appraisals, it seemed wise to adopt the Kuhnian assumption regarding indeterminacy of theory choice. Kuhn (1977) acknowledged that choice between theories in science depends on such traditionally recognized criteria as accuracy, consistency, scope, simplicity, and fruitfulness. However, he also claimed that theory choice is indeterminate both because the criteria themselves may be imprecise and because individuals may weigh such values differently to resolve possible conflicts between them. If theory choice in science thus involves “idiosyncratic factors dependent on individual biography and personality” (Kuhn, 1977, p. 329), this could be expected to be even truer for students' appraisals of theories, not least in a “softer” field such as creativity studies.

In the present study, I chose to work with higher-education students majoring in musicology and music education. Students of musicology are rarely engaged as participants in studies of creativity, but here they were chosen in order to cover a wider range of active musical interests and creative attitudes, also potentially differing from those of pre-service music teachers. While such individual differences might affect the appraisal of theories, it also seemed relevant to ask whether the theories might indeed be differently evaluated in different musical contexts. Based on the review above, I assumed that some theories might encounter problems at least when applied to musical improvisation. Hence, the first research question was about the judged scope of the theories and about systematic biases in theory appraisal:

RQ1: Is the appraisal of theories of creativity in a musical context affected by (a) differences between musical target activities and/or (b) the characteristics of the individuals making the judgments?

While this question will first be addressed on the basis of quantitative ratings, such results can hardly suffice to demonstrate the complex ways in which individuals might come to favor certain theories over others. A low rating, say, does not contain information about the reasons for giving a low rating: for one of the participants, the reason might be a sense of lacking conceptual clarity; for another, it might be unsuitability to account for subjectively meaningful experiences, and so on. In order to understand the students' thinking on this level, we may study their argumentation. I thus chose to let music students write essays in which they would argue for their choice of creativity theories in a musical setting. In broad terms, arguments can be thought to be composed of claims and justifications for those claims. For instance, in Toulmin's (1958) scheme, claims are justified by “data” (i.e., facts) and “warrants” that register the legitimacy of appealing to the kinds of data in question, as well as “backing” for the warrants. The structures of student-generated arguments, too, are typically understood to consist of a claim-like component and one or more justification components, the types of which differ between analytical frameworks (see Sampson and Clark, 2008). In the present case, claims concern the suitability of a given theory to musical creativity in general or to a particular kind of musical activity. Justifications, in turn, might conceivably differ between individuals. For instance, some students might refer to their personal experiences as support while others could rely on more abstract reasoning. In the present context, I will forgo trying to explain such individual preferences in argumentative style. Assuming a range of justificatory strategies, the second research question addressed instead the possibility that these strategies might be context dependent:

RQ2: In applying theories of creativity to music, are students' argumentative strategies dependent on the theories in question and/or on the musical target activities?

Studying music students' theory appraisal should help toward a better understanding of the relationships between theories of creativity and musical practitioners' views. My working assumption was that musically active adults are not only able to channel many of their implicit conceptions through the conscious application of scholarly theories but that they could also offer potentially valuable criticism regarding such theories. Being embraced by higher-education music students might not, of course, be necessary for a good theory of musical creativity, but a potential lack of such acceptance should at least raise interesting questions about the nature of the theories. The third research question thus addressed the fate of the theories in students' hands:

RQ3: Which theories of creativity do the students find particularly suitable for musical activities, and which ones do they find problematic in this respect?

Notice that a relative theory skepticism or a theory eclecticism on the part of the students could be potential answers to this question. However, eclectic choice of theories, in particular, would also raise new questions about the supposed nature of theories and how they are to be used and chosen. Thus, the final research question was an overarching one:

RQ4: What are students' dominant conceptions of theories and theory choice?

## Methods

### Research Strategy

The overall research strategy was based on the idea that different aspects of students' theory appraisal could be captured by different methodological approaches. First, the influence of target activities and individual characteristics on theory appraisal (RQ1) was addressed in a quantitative approach, working with theory ratings. Second, the dependence of argumentative strategies on theories and target activities (RQ2) was approached in a mixed-method approach in the sense that qualitative and quantitative aspects of the analysis were integrated before drawing conclusions (see Bazeley, 2018). Third, students' views regarding the suitability of the particular theories (RQ3) were addressed in a multimethod approach in which both quantitative and qualitative results provided complementary results that could be integrated while drawing conclusions (Bazeley, 2018). Finally, the question about students' dominant conceptions regarding theory choice (RQ4) could only be addressed by way of a philosophically oriented interpretation of the whole set of empirical results. Thus, the final research question will be postponed to the discussion.

### Participants

The participants were 47 Finnish university students of music, with a mean age of 27.4 years (sd = 6.8). They were majoring in either musicology (18 females, 16 males) or music education (8 females, 5 males). They took part in the study while taking an advanced course in musical creativity, either in 2013 (22 participants) or in 2015 (25 participants). Thirty-five of the students were at least in their fourth year of university studies, and 18 of them had a previous conservatory degree. The participants reported an average of 15.3 years of active musical experience (playing or singing; sd = 7.6), and they reported to play 3.2 different musical instruments, on average (sd = 2.0). On a scale between 0 (“not at all experience”) and 5 (“very much experience”), their reported average experience in composing (M = 3.2, sd = 1.3), improvisation (M = 3.0, sd = 1.3), working with music technology (M = 3.0, sd = 1.2), and teaching music (M = 2.6, sd = 1.7) were all above the midpoint of the scale. They did not have much experience in instrument making (M = 0.5, sd = 0.6). In assessing their own experience of making music in various genres on similar scales between 0 and 5, they reported most extensive experience in the areas of popular music (M = 3.7, sd = 1.2) and Western classical music (M = 3.2, sd = 1.7), while most had less experience from jazz (M = 1.9, sd = 1.2) and folk music (M = 1.8, sd = 1.4).

### Material

In order to avoid personal biases in the choice of theories, I selected the chapter “Theories of Creativity” (Kozbelt et al., 2010) from the first edition of the *Cambridge Handbook of Creativity* as the basis of the study. The chapter offers a balanced review of general (non-domain-specific) theories of creativity, emphasizing theoretical pluralism. The first main section of the chapter discusses classifying and comparing theories, categories of creative magnitude (e.g., “Big C/little c” creativity), the so-called four Ps of creativity (process, product, person, and place), and related schemes. The second main section includes 10 subsections, introducing the reader to as many categories of more specific theories presented in the research literature.

From each of these 10 subsections, I selected one theory that appeared to be most thoroughly described. As an exception, two theories were selected in the section “Stage and componential process theories” (reflecting both of these two aspects), and the theory of Divergent Thinking got to represent two of the subsections in which it figured centrally. For each of the chosen 10 theories, I extracted what I interpreted as core descriptive sentences regarding the basic content of the theory, removed references to literature, and substituted theorists' names with general descriptions (e.g., “some theorists”). If required, sentences from different parts of the original text were patched together, adding some words where needed. In each case, the goal was to achieve a brief, coherent description, keeping as close as possible to the handbook text. The descriptions are shown in Appendix 1, complete with quotation marks to indicate the original passages. Square brackets indicate words or phrases added to the original wordings for clarity, or places where references or other words have been removed from the citations. For presentation in the study, the quotation marks and square brackets were removed, arriving at 10 concise theoretical summaries. These ranged from 2 through 6 sentences, depending on how much material was available in the handbook text.

### Procedure

In two separate years, two groups of music students took part in a course on musical creativity. In the beginning of the course, they were given the assignment to read the original handbook chapter by Kozbelt et al. (2010), after which they took part in one of two 1-h sessions in which the chapter's contents were discussed. In facilitating the group discussions, I strived to refrain from all value judgments regarding the theories and avoided providing explanations beyond what was said in the handbook chapter. Instead, I attempted to ensure that all 10 theories were discussed, encouraging the participants to apply the theoretical ideas to their own musical experiences. The students were oblivious to the later assignment in which the theoretical summaries would be used.

During the following 3 months, the students took part in 10 classes focusing on various aspects of musical creativity on the basis of readings from different areas of music research. The obligatory readings covered topics in music history, including the myth of genius (Higgins, 2004), originality and plagiarism (Buelow, 1990; Winemiller, 1997), and theories of musical influence (Straus, 1990; Yudkin, 1992); readings in improvisation from ethnomusicological (Nettl, 1974; Nettl and Riddle, 1998), pedagogical (Tafuri, 2006; Huovinen et al., 2011), and cultural perspectives (Lewis, 1996; Prévost, 2004); issues of musical creativity and mental health (Nettle, 2001); social aspects of musical creativity (Frith, 2012; Littleton and Mercer, 2012); empirical research on creativity in musical performance (Williamon et al., 2006; Clarke, 2012); and philosophical aspects of the creative experience (Huovinen, 2011). Chapters from the textbook on creativity research by Runco (2007) were recommended for optional readings throughout the course, but general theories of creativity were not in focus during the class discussions during this period. The course also introduced the notion of conceptual ideation through a practical exercise in which the students created and wrote up “ideas for making music in a new way.” Students' written ideas—ranging from plans for new instruments through compositional algorithms to ideas for social organizations of musical life—were shared with and evaluated by other participants in the group.

Twelve weeks after their group discussion on Kozbelt et al. (2010), the students participated in one of two 105-min class sessions in which they received a questionnaire incorporating the 10 theoretical summaries edited from the handbook chapter. The students were instructed to carry out three tasks. First, they were asked to read the theoretical summaries and to evaluate the theories on 6-point Likert scales for suitability in accounting for (a) musical improvisation, (b) musical composition, (c) performance of composed music, and (d) creating ideas for making music (henceforth: “ideation”). It was explained that they should assess to what extent each of these areas of creativity would be describable, researchable, and/or understandable through the given theories. The four target activities were not further defined; instead, it was hoped that the students' varying musical backgrounds and experiences would be reflected in a wide range of understandings concerning such activities.

Second, the students were asked to choose 1–4 “best theoretical perspectives” that “best correspond to your own thoughts about what is central for musical creativity.” Their task was to write an essay justifying their choice of theories, paying attention to whether various forms of musical creativity might require different theoretical perspectives. The students were also encouraged to reflect on possibilities for research in connection with the theories, to discuss problems in applying the theories to music, and to suggest refinements to the theories for the purpose of using them in musical contexts. No instructions were given concerning the lengths of the essays.

In order to assess the possible effects of personality on theory choice, the Five-Factor Model of personality (Digman, 1990), also known as the “Big Five” (Goldberg, 1993) was assumed as a starting point. The students filled out background questionnaires as well as the “Short Five” personality test (Konstabel et al., 2012), measuring the 30 facets of the Five-Factor Model with 60 comprehensive single items (positive and negative statements intended to match expert descriptions of the constructs). The Finnish-language version of the test used here has been shown by Konstabel et al. (2012) to have good to excellent congruence with the structure of the standard NEO Personality Inventory-Revised (see Costa and McCrae, 2014).

All students received course credit for the assignment. It was explained that apart from the course assignment, they could freely choose to allow their responses to be used anonymously in the author's research, and that in so doing, they could withdraw from the study at any time. Six students did not agree to participate, and their responses were removed from the data reported here. The remaining 47 participants gave their informed consent in written form. The background information reported above as well as the results concern these 47 participants. Institutional guidelines for ethical practice were followed throughout the study.

### Analysis

Quantitative analyses of the ratings were carried out in the *R* environment for statistical computing (R Core Team, 2019), using the package “psych” for principal component analysis of theory ratings (Revelle, 2019). Linear mixed-effect models for the ensuing principal components were built using the “lme4” package (Bates et al., 2015), and estimated marginal means were produced using the “emmeans” package (Lenth et al., 2019). Other quantitative methods involved in the analysis of ratings were Kruskal–Wallis and Mann–Whitney *U*-tests, as well as the Pearson correlation coefficient.

The participants wrote their essays in Finnish, apart from two bilingual participants who chose to write in English. The hand-written essays were first typed into digital format. Their length ranged between 1,752 and 8,892 characters (spaces excluded), with a mean of 3,672 characters (SD = 1,571) per essay.

A content analysis of the essays was carried out by coding them in the program NVivo (QSR International Pty Ltd, 2018). This involved three separate content codings, initially marking passages for (1) each of the 10 theories of creativity and (2) each of the four target activities mentioned in the task instructions (improvisation, composition, performance, ideation). In both of these cases, coded passages could range from parts of sentences up to longer paragraphs (and in rare cases, even the entire essay, when the same construct had been given a longer, continuous discussion), and several overlapping codes could be used. Finally, the text was coded for (3) argumentative content, based on the idea that arguments consist of claims and justifications (e.g., Sampson and Clark, 2008). Claims, in this case, were assertions concerning the suitability of a given theory to account for musical creativity (or some form thereof). Quantitative aspects of the essay responses were analyzed in *R* using χ^2^ tests for the equality of proportions and hierarchical cluster analysis (using the complete linkage method).

## Results I: Ratings

### Theory Ratings and Theoretical Dimensions

**Figure 1:**
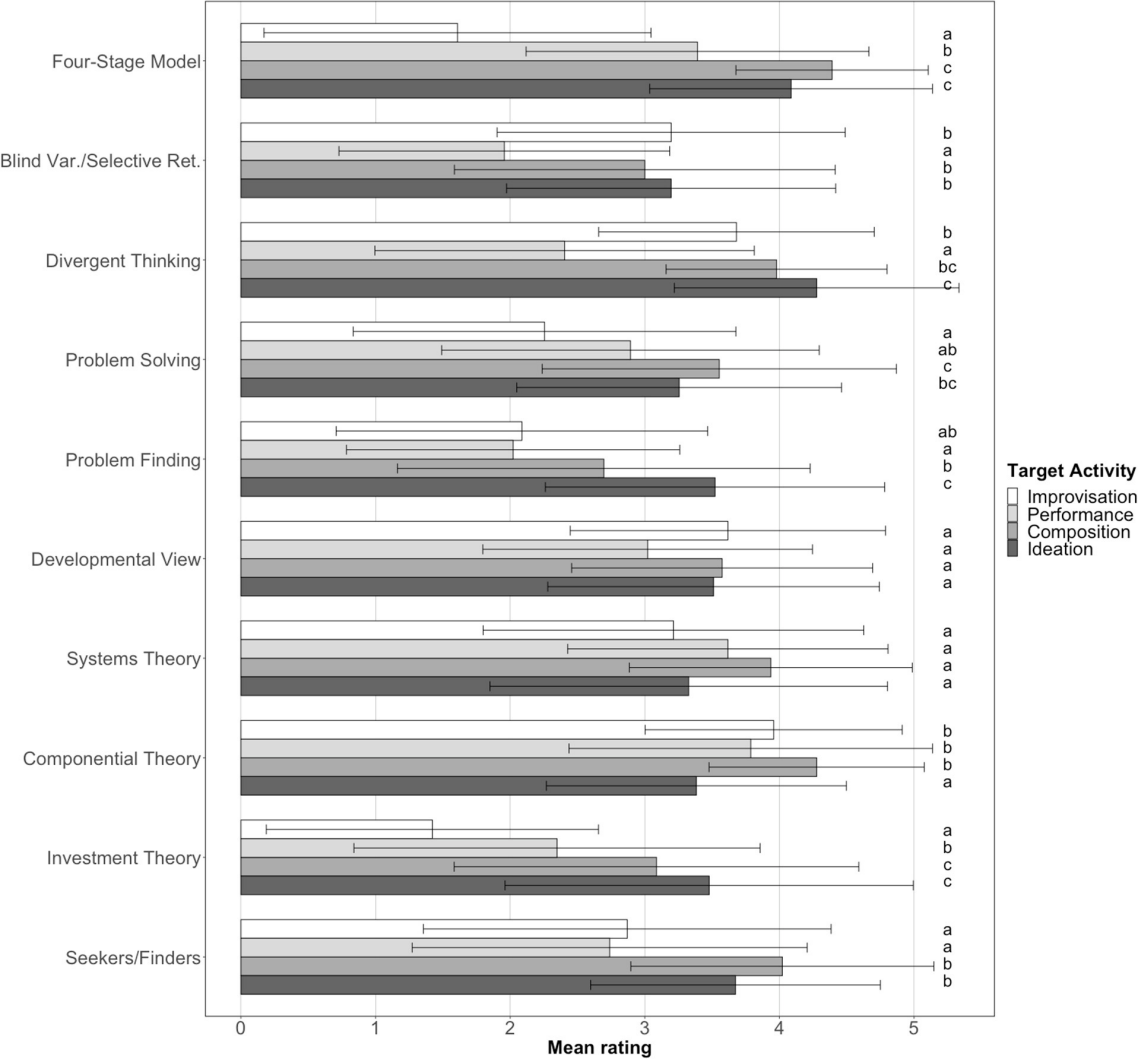
Mean ratings of the 10 theories for the four musical target activities, with standard deviation error bars and compact letter display of pairwise comparisons. Within each theory, activities with the same letter did not differ significantly (*p* < 0.05) from one another in their ratings.

The highest mean rating was obtained by Amabile's (1996) Componential Theory (M = 3.9, sd = 1.1), closely followed by Divergent Thinking (M = 3.6, sd = 1.3) and Systems Theory (M = 3.5, sd = 1.3). However, for eight of the theories, Kruskal–Wallis tests with *p*-values adjusted for multiple comparisons showed significant (*p* < 0.01/10) differences in ratings between the four target activities. These differences are shown in Figure 1, using compact letter displays to indicate pairwise comparisons between activities (Dunn's tests). Generally, differences between target activities were smallest for theoretical summaries making no claims about the creative process (Developmental View, Systems Theory, Componential Theory), whereas some other theories were deemed relatively unsuitable either for musical improvisation or performance (or both). In particular, the Four-Stage Model and the Investment Theory (Sternberg and Lubart, 1991; Sternberg, 2012) seemed least acceptable as accounts of musical improvisation.

Each theory was rated by the participants four times, relating it to each of the four musical target activities; thus, for each theory there were 188 ratings. It would be unlikely that all such sets of ratings would be completely independent of one another. Instead, we could expect to find a smaller number of basic theoretical dimensions along which several theories receive similar ratings in a number of musical contexts. In order to condense the rating data to such dimensions, a principal component analysis (with varimax rotation) was carried out, extracting four components (with eigenvalues larger than 1). The resulting analysis is seen in Table 1, showing loadings above 0.4.

**Table 1:** Principal component analysis of participants' ratings for the 10 theories (with varimax rotation).

	**Orderly Process**	**Strategic Divergence**	**Darwinian Divergence**	**Socio-Cognitive System**	**Communality**
Problem Solving	0.77				0.60
Four-Stage Model	0.71				0.65
Seekers/Finders	0.66				0.47
Investment Theory		0.76			0.61
Problem Finding		0.60			0.47
Blind Variation/Selective Retention			0.79		0.63
Developmental View			0.64		0.43
Divergent Thinking		0.54	0.50		0.57
Componential Theory				0.77	0.72
Systems Theory				0.76	0.72
Sum of squared loadings	1.627	1.614	1.430	1.225	
Proportion of variance	0.163	0.161	0.143	0.122	
Cumulative variance	0.163	0.324	0.467	0.590	

I have tentatively named the four emerging theoretical dimensions according to salient common ideas shared by the theories with high loadings on these dimensions. The first component could be interpreted as focusing on an *Orderly Process*: the two theories with highest loadings on this component (Problem-Solving and Four-Stage Model) emphasize an orderly thought process through which an idea or solution is reached. The inclusion of Seekers/Finders in this component may reflect the fact that both types of creators were accounted for by their characteristic working processes. In other words, it is the emphasis on process rather than either of the types of creators that groups this theory with the two others. The second component, *Strategic Divergence*, appears to center on making a strategic contribution by investing in a new idea (Investment Theory) or a new problem (Problem Finding) that diverges from commonplace solutions in its originality (Divergent Thinking). Notice that all of these three theories require the creative achievement to be assessed in its historical dimension (as “H-creativity”; Boden, 1990) or in terms of some other comparison to standard achievements. The third component also involves Divergent Thinking, but this component could be called *Darwinian Divergence*, as it is dominated by ideas of Blind Variation/Selective Retention and development through environmental influences (Developmental View). Finally, the fourth component highlights creativity as a *Socio-Cognitive System*: it includes theories that describe creativity as involving a field of gatekeepers (Systems Theory) or as an interaction between dispositional, cognitive, and social aspects (Componential Theory).

### Target Activities and Individual Characteristics

Equipped with a more condensed account of the theoretical dimensions, we may reformulate the first research question and ask: does the appreciation of the four theoretical dimensions depend on types of musical activities considered and/or on the evaluator's own individual characteristics or musical background? To address this question, the principal component scores were normalized between 0 and 1, yielding four synthetic variables, one for each theoretical dimension.

None of these variables appeared to be significantly affected by participants' gender or the participants' major subject of study (Mann–Whitney *U*: all *p*s > 0.1). To study the possible effects of other background variables, I constructed linear mixed-effect models for each of the principal component scores, taking participant as random effect. In each model, musical activity was included as a fixed effect. To choose other fixed effects, Pearson correlation was first used to screen the participants' background variables for associations with the theoretical dimensions (for the linear modeling, these variables were interpreted as interval variables). Apart from participants' age, year of study, years of musical activity, and the five facets of the Short Five personality test (N, E, O, A, C), the variables considered included self-evaluations (on a 6-point scale) regarding experience in composition using traditional notation, composition with other means, improvisation, music technology, and music teaching, as well as playing music in the areas of classical music, popular music, jazz, and folk music. The preliminary correlation analysis revealed only very few potentially relevant background variables, most notably composition experience (using traditional notation) and jazz experience.

Using a likelihood-ratio approach, mixed models were then constructed as shown in Table 2. (Given some missing data, there were initially 175 observations for each model. On the basis of Q–Q plots, one extreme observation was further discarded in the model for Darwinian Divergence, and two in the one for Socio-Cognitive System.) The results show that the type of musical activity had a highly significant effect on each theoretical dimension. Moreover, the background variable of composition experience improved the models for both Strategic and Darwinian Divergence. Finally, jazz experience improved the model for the Socio-Cognitive System dimension. No other fixed effects or interactions could be used to improve the models.

**Table 2:** Construction of mixed models for the four dimensions of creativity theories.

		**Model fit**			**Likelihood-ratio rests**		
		**Marg**. ***R**^**2**^*	**Cond**. ***R**^**2**^*	***AIC***	**χ^2^**	**df**	***p*** **(> χ^2^)**
Orderly Process	Random effect	0	0.058	−40.81			
	Target activity	0.299	0.453	−106.49	71.68	3	<0.001^***^
Strategic Divergence	Random effect	0	0.263	−98.71			
	Target activity	0.249	0.594	−170.71	77.99	3	<0.001^***^
	Composition exp.	0.291	0.594	−173.04	4.33	1	0.037^*^
Darwinian Divergence	Random effect	0	0.219	−83.85			
	Target activity	0.297	0.609	−168.75	90.90	3	<0.001^***^
	Composition exp.	0.351	0.609	−172.93	6.18	1	0.013^*^
Socio-Cognitive System	Random effect	0	0.317	−63.43			
	Target activity	0.093	0.441	−83.27	25.83	3	<0.001^***^
	Jazz experience	0.145	0.442	−86.15	4.88	1	0.027^*^

Significance levels:

*** *p < 0.001*,

* *p < 0.05*.

**Figure 2:**
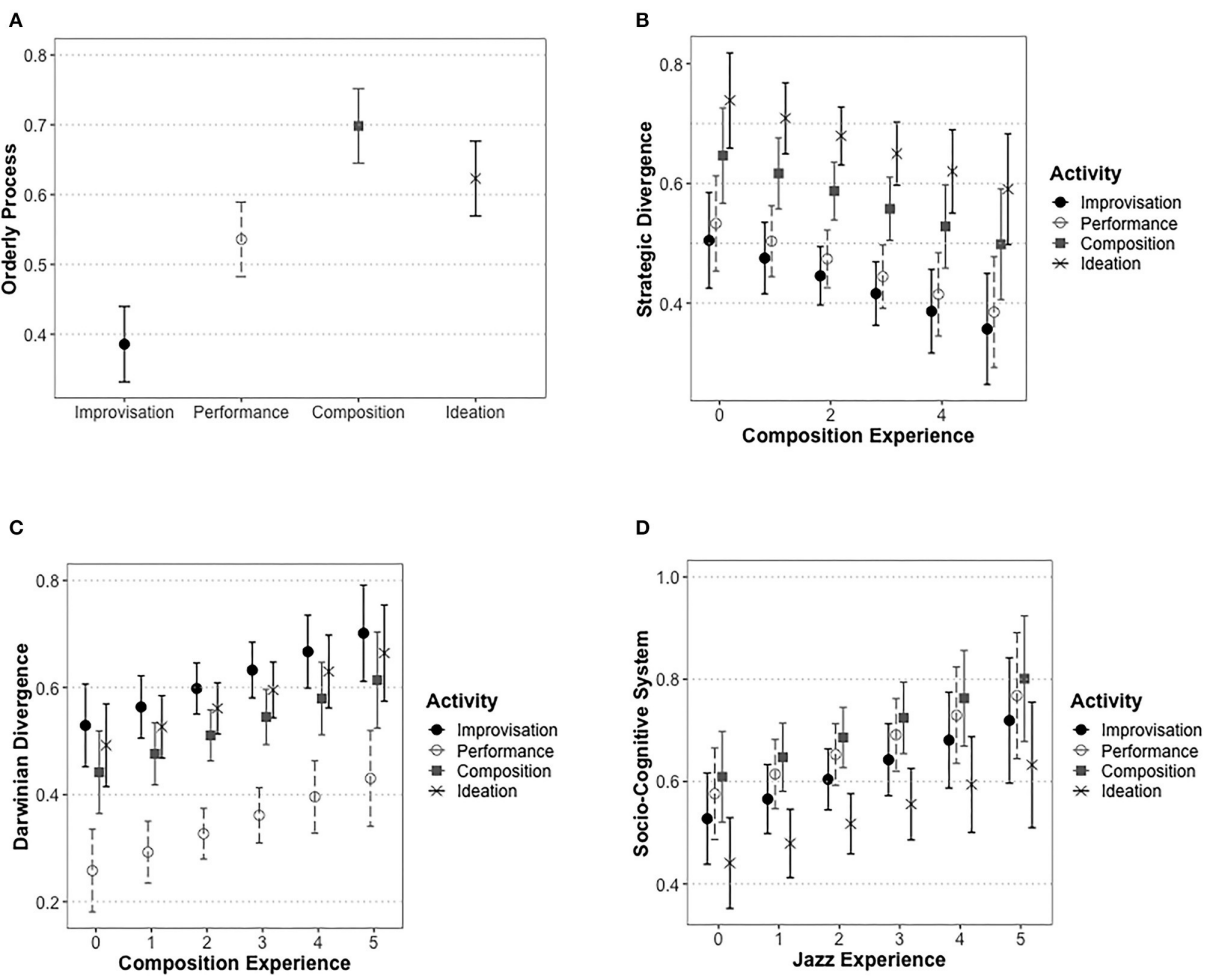
Appreciation of the four theoretical dimensions: predicted values from linear mixed models (with 95% CIs). **(A)** Orderly Process. **(B)** Strategic Divergence. **(C)** Darwinian Divergence. **(D)** Socio-Cognitive System.

Predicted values from the four models are plotted in Figure 2. Beginning with the effects of musical activity, we may note that the first two theoretical dimensions appeared especially suitable for musical activities that tend to take place outside of performance situations. Thus, emphasis on Orderly Process was especially favored in connection with composition and ideation, whereas it was found less suitable for performance and, especially, for improvisation (Figure 2A). Similarly, emphasis on Strategic Divergence mostly emerged for ideation and composition, whereas such a perspective was not as favored for performance or improvisation (Figure 2B). The two last theoretical dimensions show a different picture: in both cases, one of the musical activities stood out as being the least suitable target to be accounted for in the terms in question. On the one hand, Darwinian Divergence appeared least suitable for musical performance (Figure 2C). On the other, the approaches appearing under the Socio-Cognitive System dimension were found least suitable for ideation (Figure 2D).

As was made clear above, among the background variables only self-reported composition experience and jazz experience were found to improve the models for the theoretical dimensions. As seen in Figure 2B, composition experience decreased the appeal of Strategic Divergence. Thus, even if the strategically rational notions of investment and problem finding might be seen as compatible with (modernist notions of) musical composition, our compositionally active participants seemed opposed to such ideas. Moreover, the lack of interaction with musical activity indicates that their relative resistance to Strategic Divergence not only concerned the activity of musical composition as such, but it appeared across the board for all musical activities. By contrast, composition experience also seemed to make the participants more willing to approach all musical activities as Darwinian Divergence (see Figure 2C). Such results suggest that compositional experience may have familiarized students with free, “blind” generation of musical ideas and materials, unhampered by strategic aims. Similarly, Figure 2D suggests that receptivity to ideas of creativity as a Socio-Cognitive System was furthered by participants' jazz experience.

## Results II: Argumentation

### Applying Theories to Activities

According to the task instructions, the participants were to defend the “best theoretical perspectives” in their essays. As shown in the left column of Table 3, almost half of the 47 participants chose to defend the Four-Stage Model, but Divergent Thinking and Systems Theory were not left far behind. Overall, the numbers of participants choosing to defend a particular theory differed significantly between the 10 theories [χ^2^(9) = 55.24, *p* < 0.001]. The three theories that above received the lowest mean ratings were also here least often chosen to be defended. Hence, the two theories that formed the core of the theoretical dimension of Strategic Contribution (i.e., Investment Theory and Problem Finding) were both only chosen to be discussed in four essays. Likewise, even though Blind Variation/Selective Retention was above seen as the central theory for the dimension of Darwinian Divergence, here the theory was only defended by two participants. Notice, then, that while Divergent Thinking in the rating task tended to be enhanced by the striking notions of investment, problem finding, or blind generation, none of the latter ideas were found very appealing for music as such.

**Table 3:** Numbers of participants defending the theories in their essays and applying them to the four musical activities (*N* = 47).

**Theory**	**Chosen to be defended**	**Applied to target activities**			
		**Composition**	**Improvisation**	**Performance**	**Ideation**
Four-Stage Model	22	20	13	15	11
Divergent Thinking	20	11	14	10	14
Systems Theory	19	12	10	11	7
Componential Theory	15	8	10	10	4
Developmental View	14	8	12	9	6
Seekers/Finders	13	11	10	6	6
Problem Solving	6	5	3	3	2
Investment Theory	4	3	2	3	3
Problem Finding	4	0	2	1	2
Blind Variation/Selective Retention	2	2	2	0	0

The essays were first coded for passages concerning the 10 theories as well as for the four target activities. Using the matrix coding function of NVivo, I thereafter analyzed the overlaps between these two sets of codes (manually correcting the numbers of participants if a single participant showed multiple overlaps between the same codes). The four last columns in Table 3 show the numbers of participants mentioning any of the 10 theories in conjunction with the four kinds of musical activities. Three of these distributions did not significantly differ from the “chosen to be defended” column, but in the case of composition, there was a significant difference [χ^2^(9) = 20.85, *p* < 0.05/4], largely due to the relative success of the Four-Stage Model as an account of compositional work, over and above the other theories.

### Argumentation for Theories

The third coding of the essays concerned argumentative content and was carried out in a bottom-up fashion, based on the idea that claims concerning the suitability of theories could be justified in various ways—by appealing to rational reasoning, authority, one's own experience, etc. After an initial coding round, the emerging classes of statements (including longer coherent passages) were reread, attempting to clarify the distinctions between argumentative categories. For instance, the boundary between *generalized illustrations* and (a preliminary category of) *examples* was sharpened by restricting the latter to *particular examples* that focused on individual persons (e.g., Miles Davis) or other historically particular circumstances (e.g., the performance tradition of Russian violinists). Generalized illustrations of the theories, in turn, were lacking in such anchoring to particular individuals or circumstances (e.g., “Perhaps this sort of creative process can be found behind many musical instruments: someone has found a mechanism or approach almost by chance and started to develop it” [participant P12 on Problem Finding]). During this honing process, some preliminary categories were combined: for instance, statements that had first been taken separately as *individually chosen premises* for theorizing (e.g., “the theory should address factors related to the temporal duration of the process such as motivation and environment” [P14]) were combined into the category *theoretical reasoning* that also included more developed conceptual arguments (see an example in Appendix 2).

The final coding scheme included four categories of *claims* and nine categories of *justifications* (see Appendix 2). Apart from the central *positive claims*, suggesting the suitability of a theory to music or some musical activity, claims also included corresponding *negative claims*, and *theory descriptions* that simply explained the theories or highlighted some of their aspects, as well as *free generalizations* that did not have a clear justificatory function. Justifications, in turn, comprised *generalized illustrations*, appeals to *personal experience, particular examples, values*, and *authority*, as well as the use of *theoretical reasoning*. In addition, some passages were interpreted as regarding *theoretical complementation* (i.e., relating several theories to one another to strengthen the overall account), as suggestions concerning *application to research* (often stating a personal research interest involving the theory), or as *problematization* in which a given theory was more substantially argued against (instead of a simple negative claim concerning its suitability). This framework necessarily involves some interpretative leeway both in the boundaries between the categories and, especially, in how particular statements were demarcated and how separate arguments were individuated in the texts. In order to alleviate the latter problems, the quantitative parts of the following account are simply based on numbers of participants that were found to use given argumentative means somewhere in their essays in conjunction with given theories or given types of musical activity.

The distribution of the argumentative means in Table 4 shows that most (45/47) of the participants made direct positive claims regarding the suitability of theories in musical contexts and that the majority (40/47) argued for their views using at least some generalized illustrations. For a simple example of this argumentative strategy, consider the following extract from a musicology student's essay. A positive claim concerning the suitability of the Systems Theory is directly followed by a generalized illustration that simply states the main aspects of the theory in a musical setting:

**Table 4:** The distribution of claims and justifications in the participants' essays (numbers of participants applying a given argumentative means for discussing a given theory).

		**Four-Stage Model**	**Divergent Thinking**	**Systems Theory**	**Componential Theory**	**Developmental View**	**Seekers/Finders**	**Problem Solving**	**Problem Finding**	**Investment Theory**	**Blind Var./Selective Ret**.	**Total Participants**	**χ^2^**
Claims	Positive claim	21	14	17	14	14	11	3	6	3	3	45	17.66
	Theory description	17	14	12	11	10	6	4	2	2	1	38	13.66
	Free generalization	6	2	10	4	6	4	2	1	1	1	24	12.89
	Negative claim	2	1	1	0	1	1	1	3	0	0	11	11.57
Justifications	Generalized illustration	19	13	12	8	6	7	5	4	3	2	40	10.02
	Problematization	15	7	7	4	6	4	5	3	1	2	33	9.36
	Theoretical complementation	7	7	6	6	3	6	3	3	1	1	25	3.37
	Appeal to personal experience	13	3	2	1	3	5	2	0	0	0	21	27.21^*^
	Appeal to values	2	10	2	5	0	4	0	5	2	0	20	28.18^*^
	Appeal to authority	3	4	5	2	5	1	1	1	1	0	18	6.81
	Appeal to particular example	2	1	6	0	2	1	0	0	2	0	16	22.27
	Application to research	4	3	4	3	5	3	1	1	0	2	15	5.21
	Theoretical reasoning	1	6	4	4	1	0	3	2	0	0	14	14.22
	Total participants	26	23	20	18	18	15	11	10	6	6	47	

Significance levels:

* *p < 0.05/13*.

[*Positive claim*:] The theory of creativity as a system also well describes working out ideas for music (and composition). [*Generalized illustration*:] Producing a musical idea requires knowledge about the domain, a person who knows things and can for instance develop a new instrument, and an area in which she can further her invention with the help of other colleagues. These, in turn, decide whether the idea is good enough to be published. (P15)

The passage following the positive claim would otherwise be labeled a *theory description*, but the idea of developing a new instrument turns it into a musical illustration of the theory—albeit a highly generalized one. Such generalized illustrations were used for all theories approximately in equal proportion to how often each theory was taken up by the participants. On each row of Table 4, the numbers of participants applying the argumentative means in question to the 10 theories has been compared to the total numbers of participants discussing the 10 theories in their essays. On each row, the χ^2^ test thus indicates whether the distribution of a particular argumentative means differed significantly from the distribution on the bottom row of the table. (The α levels have been adjusted for multiple comparisons: α = 0.05/13). Significant differences from the bottom row were found in the appeals to personal experience and in appeals to values.

First, we may note that more than half of the participants who made references to their own personal experiences did this (at least) in discussions of the Four-Stage Model. Some of them suggested that their own musical orientation (“toward the production side,” P14) or, in more essentialist terms, their nature as a “seeker” (P31) or “a logical person” (P13) made the Four-Stage Model relevant to their creative experiences. More often, participants simply mentioned that “the four stages of the theory are easy to discern in my own work” (P27; similarly P2, P9, P26, P32, P43). More specific applications were mentioned as well. One participant referred to having become convinced of the theory by listening to a particular musician (P46), and two mentioned their experiences of creating ideas for music (P36, P45). One of these, a young student of musicology, chose to write her entire essay on the virtues of the Four-Stage Model, explaining each of the four target activities on this basis. She fluently described her own experiences of incubation and illumination in two of the activities that were familiar to her and gave briefer accounts for the two others for which she apparently lacked personal experience. In her longer accounts, she described how ideas for musical compositions or arrangements had just come to her while sitting on a bus or while listening to the radio. In the more experienced end, a 34-year-old student of musicology who reported playing six instruments and working as a musical playschool teacher described her own work with children using the Four-Stage Model. She described a group process of creating music with children in which she felt that her own creative achievement was enhanced by giving the children time to incubate:

I create a lot of teaching materials: children's songs and rhymes as needed. Some of these come in a moment, but my best products I have managed to get notated and into heavy use through exactly this sort of many-staged process. […] This fall, in my work at a music playschool, I have tried to give more time to the children's own creative ideas and thoughts. I mean for instance when we made a little Christmas musical with one group. “Giving time” [i.e., incubation] consisted in returning to the ideas in many lessons—in refining and developing them over a longer period of time. (P29)

By contrast, other theories that were often discussed received very few justifications by appeal to personal experience, despite the many positive claims in their favor. As regards Divergent Thinking, two participants briefly mentioned how the theory “corresponds to my experience of improvising” (P15) or how “in my experience, this type of thinking works and yields good results in musical ideation” (P16), while one somewhat unclearly argued for the theory based on her predilection for working processually (P34). For the Developmental View, one of the students simply referred to her “personal experience” (P32), and two others gave examples of their family background that they did not seem to take as equivocal support for the theory. One of these participants wrote that she, as a musical person, was from no musical family herself, but that she had nevertheless been supported in her musical hobby when she had come upon the idea herself (P42). Another found support for the theory in that “I was never encouraged to improvise, which I believe to have affected my current [negative] attitude [toward improvisation]”; at the same time, she also used a counterexample from her family: “Exceptions always exist, and even providing creative space does not always lead to musical creativity. This happened to my sister, who went to piano lessons, children's choir, and to music theory and solfege lessons, but does not do music anymore in any way” (P39).

Turning to the Componential Theory, the only appeal to experience consisted of a single clause in which the participant referred to her experience of rating the theories earlier during the session (rather than to her prior musical experiences). Finally, in the case of Systems Theory, only two participants appealed to their personal experience, both of them using this less as an argument in its own right than to highlight the differences in how creative products could be received in different historical circumstances (e.g., because of their technological underpinnings, “the ideas for music production that we brought to class [in another course assignment] could have been received differently in another era” [P41]). In such cases, one might even contest the interpretation as appeals to personal experience, but the point is that for these theories, no clearer appeals to personal experience were made at all. This is perhaps understandable: particular, lived experiences as such may not be enough to support ideas of multicomponent systems, theories based on statistical observations (Developmental View), or ones that otherwise require judging the divergence or usefulness of ideas or products from an “outsider” perspective.

The other argumentative means with a unique distribution of participants between the theories was appeal to values (see Table 4). Here, the two favorite theories in the essays showed a picture quite opposite to what was above seen with personal experiences. While the Four-Stage Model was only twice justified by normative appeal to values (e.g., “A fine result […] requires preparation, mental processing etc.” [P44]), half of the 20 participants appealing to values used this strategy in connection with Divergent Thinking. According to these participants, musical ideation (P19) or composition (P34) can be “at its best” when the creator works divergently: “in musical ideation, diverting from mainstream thought can be essential” (P19), and “new ideas are needed for music to be reformed” (P47). A 31-year-old popular music guitarist, close to graduation in musicology, went into more depth about the “essential” role of divergent thinking in the creative process:

There is the danger that you cannot decide which idea to start working on, and that you instead even-handedly throw around different ideas. In order to progress, it is essential that you have an initial idea that is then subjected to incoherence. […] It is not so important what the original idea was, but it is important to begin from somewhere. (P12)

Some of the participants also showed awareness of how their own aesthetic values may have affected their attitude toward Divergent Thinking: “I may have chosen this theory, because I myself value creative ideas that are also practical” (P43). In other cases, the authors anchored their own value judgments in beliefs about other people's aesthetic values: divergent thought can thus be “important if the goal of the composers is to get credit for their creativity in a community” (P10). Indeed, theorizing about Divergent Thinking often seemed to require addressing the experiences of other people: “the divergent thinker should also show some practical thinking, so that the excessive originality of ideas does not begin to erode their value: […] when originality transcends the understanding of the audience, the value ascribed to the work by the audience begins to descend” (P14). In the following example, another student of musicology similarly made value judgments of his own, first about divergent thought in composition and then in improvisation, each time bolstering his own value judgments (“it may be beneficial,” “may be a double-edged sword”) by referring to the aesthetic values of the public:

In composition, it may be beneficial for the composer to think divergently. This is because the public often appreciates surprise in musical works—albeit too much surprise […] in composition may also be disadvantageous. […] As in composition, divergent thinking may also be a double-edged sword in improvisation. Too much “jazzing” by, say, a dance-band guitarist may lead to falling out of the audience's favor. However, some also prefer surprisingness and unconventionality in improvisation. (P4)

### Argumentation Regarding Musical Activities

Running a similar analysis of argumentative means in connection with the four musical target activities led to a simple observation. For most argumentative means, the distribution of participants using the argumentative strategy in the four activities did not significantly differ from the overall numbers of participants discussing these activities (43 improvisation; 46 composition; 41 performance; 33 ideation). The exception was *problematization* for which the distribution of participants was heavily biased. Among the 30 respondents using problematization, 19 did this while applying theories to improvisation, whereas only 9 problematized theories in composition, 3 in performance, and 6 in ideation [χ^2^(3) = 16.57, *p* < 0.05/13]. Interestingly, 11 of the critical responses regarding improvisation were specifically about the Four-Stage Model, 10 of them pointing out problems in fitting something as fleeting as improvisation into the temporally extended framework of the theory. Some saw a problem in the first stage of preparation in which a problem is defined: “if by improvisation we mean expression taking place in a given moment of time, no first-stage problem actually exists” (P36); “you cannot prepare if you live in the moment” (P3). More often, the trouble seemed to lie in the incubation stage of the model: “there is no time for incubation in momentary discovery” (P10, similarly P3, P4, P20, P29). Along similar lines, one student of musicology remarked that applying the Four-Stage Model to improvisation would require either “running through the [four-stage] process very rapidly, leaving out certain stages, or confining oneself to only the last two stages (and thus improvisation would be ‘illumination’ or verification of what has previously been absorbed)” (P8).

While improvisation most often created problems for the Four-Stage Model, each of the other theories were problematized once or twice as applied to improvisation (with the exception of the Investment Theory that simply appeared to be ignored as irrelevant for improvisation). A heavy-metal guitarist, for instance, saw the Systems Theory as “leaving a cold view of improvisation” as it ignores “little pitch-level details” and generally “leaves in the dark the individual that is often central in improvisation” (P41). Overall, students' problematizations revealed a range of different conceptualizations regarding improvisation. Discussing the Systems Theory, a classical violinist expressed the opinion that “purely expressive improvisation […] is not even meant to be linked to a certain tradition, its products often not meant to be preserved for posterity” (P38). Quite to the contrary, a 33-year-old student of music education with multi-instrumental skills (nine instruments) and extensive improvisation experience saw Divergent Thinking as problematic for improvisation exactly because improvisation is often subject to traditional constraints (see Appendix 2). For another example of opposing views, one participant said she “feels that in improvisation a problem is not solved, but rather found” (P32), while another thought that “problem finding requires profound thinking of the matter” which is not possible in improvisation (P12). The participants' prolematizations thus show how improvisation often required stretching or reinterpreting the theories, leading the participants to different directions. One music education student in her senior year admitted that she had been unable to find “the most explanatory theory” for improvisation, because the notion of improvisation itself is slippery, lacking a clear definition. For her, this state of affairs was supported by her own musical experience: “sometimes, improvisation springs from a primitive unconscious, while sometimes it arises on the basis of certain musical models” (P43).

### Combining Theories

In their essays, the participants chose to defend an average of 2.5 theories (SD = 1.0), and the chosen combinations of theories formed relatively distinct types. A hierarchical cluster analysis of the defended theories yielded a solution in which the first main branch included essays defending the Four-Stage Model and/or Divergent Thinking (two subbranches of 13 and 17 participants corresponding to the absence and presence of Systems Theory, respectively). Given the role of personal experiences in justifying the Four-Stage Model and the role of values in defending Divergent Thinking, this branch was characterized by a “subjective” argumentative style. The second main branch de-emphasized both the Four-Stage Model and Divergent Thinking, and presented more “objective” argumentative approaches instead. Its two subbranches focused on psychological explanations, on the one hand (6 participants choosing Seekers/Finders and/or Developmental View), and systems accounts, on the other (11 participants choosing Componential Theory and/or Systems Theory).

Whether it was an artifact of the study design or not, by choosing such combinations of theories most students seemed inclined toward a certain theoretical pluralism: “creativity can be approached from many perspectives” (P44). While the need for several theories was implicit in most participants' multiple choices of theories, some of them also presented explicit arguments regarding theoretical scope: “even the most interesting theoretical perspective is not necessarily suited for understanding all areas of musical creativity” (P42). Some of the students simply argued that “by combining these perspectives according to situation, we can reach a fairly good understanding of creativity as a process” (P10), but others draw the line between musical activities. For instance: “composition and musical ideation are close to one another as phenomena, whereas improvisation and musical performance require a different theoretical approach” (P11). Theories were frequently discussed as if they allowed to “put the focus on” (P46) various aspects of a phenomenon that cannot quite be grasped in its totality in terms of one theory only. A handful of students also argued for the multicomponent or systems views on the grounds that they are “more comprehensive” than other theories (P25) and “bring together aspects from several theories” (P38).

An elaborate example of such scope argumentation appeared in the essay by a 27-year-old graduate student of musicology, known as a competent jazz pianist. After a detailed argument for the Componential Theory—itself a pluralistic combination of aspects—he argued that the “downside of a model that applies to [several] different methods of music making is that it is broad by necessity” (P5). Hence:

Due to the vast number of different tasks and methods involved [in] music making, it is my view that no one theory of creativity can describe it perfectly. Instead, the main music-making processes can be seen as being composed of a variety of smaller scale processes, and these processes have their own sub-processes. (Meanwhile, the boundaries between simultaneous processes are unclear and sometimes disappear altogether, making this an even trickier subject to tackle.) Which model we use to describe music making should depend on how close we “zoom in” on each process. The component theory works well on a broad scale, with many of the other theories being relevant with more specific processes. (P5)

Thus, in particular:

If we are to look more closely at the domain-relevant skills component in the component model of creativity, we can see that the acquiring of these skills is in itself not entirely free of creativity […]. [The acquisition] of domain-relevant skills is often a process of problem solving, which can also include its own kind of creativity […]. Likewise, divergent thinking clearly falls within the “creativity-relevant skills” component, a skill that can be used in most aspects of music making, though maybe not on a regular basis. (P5)

Accounts such as this suggest treating theories of creativity less as mutually exclusive alternatives, and more as useful ideas that may be combined in various ways in order to grasp different facets of a more complex phenomenon. In the excerpt seen above, the student goes still further, in effect working out a *reinforcement* relation between theories in which one theory provides the “rationale” for another (see Laudan, 1977).

## Discussion

In the introduction, I noted that while theories of creativity have been applied in some areas of music research, other music scholars have either ignored such theories or even opposed them with skepticism. Assuming that most theories of creativity have been meant to cover musical creativity, too, my aim in this study was to analyze musically active and relatively well-informed adults' understanding of such theories in order to gain an overview of potential stumbling blocks in this domain. In the study, higher-education music students appraised general theories of creativity as accounts of four types of musical target activities—composition, improvisation, performance of composed music, and ideation (i.e., creating ideas for making music). These activities do not, of course, cover all possible forms of musical creativity (e.g., creativity in listening) but were chosen to present some variety that might help in assessing the context dependence of the theories. Based on a classification of creativity theories in a standard reference work (Kozbelt et al., 2010), a representative sample of 10 theory descriptions was subjected to music students' ratings. The participants were also asked to choose the “best theoretical perspectives” and to argue for them in written essays. The focus on musically active people's understanding of explicitly formulated scholarly theories of creativity is rather unique, given that most research about peoples' conceptions regarding creativity has focused on informal beliefs. I will now review the findings in response to the four research questions presented in the beginning.

The first half of the first research question asked whether the judged suitability of theories would differ between the target activities. Significant differences between the activities were found for eight of the theories, and they mostly had to do with problems of accounting for musical improvisation or performance. To condense the data, a principal component analysis of participants' theory ratings was carried out. This yielded four theoretical dimensions, respectively emphasizing creativity as an Orderly Process, as Strategic or Darwinian Divergence, and as a Socio-Cognitive System. Linear mixed models showed each of these dimensions to be dependent on the target activities. The dimensions of Orderly Process and Strategic Divergence, in particular, were favored when accounting for composition and ideation, but not so much in the contexts of performance and improvisation. The dimension of Darwinian Divergence, dominated by the Blind Variation/Selective Retention theory (Campbell, 1960; Simonton, 1997, 1999, 2010, 2015), was found least appropriate for the performance of composed music. These results were complemented by how the participants in their essays chose somewhat different theories to account for various target activities. In particular, Wallas's (1926) Four-Stage Model of creativity emerged as a particularly suitable way of accounting for composition. Indeed, most of the participants who chose to defend the Four-Stage Model in their essays defended it at least as an account of composition. This resonates with some previous research in which the Four-Stage Model has been used to account for both music students' (Burnard and Younker, 2004; Chen, 2012) and professional composers' actual compositional processes (Katz, 2012; Katz and Gardner, 2012). At the same time, the theory ratings indicated appreciable problems in applying the Four-Stage Model to improvisation. All in all, the results indicate that the students viewed the theories as relatively context-dependent, and in this sense, not as very “general” theories. Based on the ratings, the exceptions seemed to be the Developmental Theory, Systems Theory, and, perhaps, Componential Theory, all of which received high ratings across the target activities. Other theories, however, seemed to encounter problems especially with the in-time processes of musical performance and/or improvisation.

The latter half of the first research question addressed whether theory appraisal would also be influenced by the “characteristics of the individuals who make the choice” (Kuhn, 1977, p. 324). In this regard, most background variables, including gender, personality, and years of musical activity, showed no systematic effect on participants' ratings. However, composition and jazz experience apparently affected their views. On the one hand, participants' receptivity to creativity as a Socio-Cognitive System was furthered by their experience of playing jazz music. This could mean that experience in improvisatory music-making is associated with the emphasis of domain-specific knowledge and skills (mentioned in both of the relevant theoretical summaries), with awareness of the relevance of task motivation, and with understanding of how creative actions are received by other members of the field. On the other hand, composition experience decreased participants' approval of creativity as Strategic Divergence but increased their approval of the dimension of Darwinian Divergence. A possible interpretation would be that solitary compositional work may have accustomed students to thinking about creativity as playful engagement with musical materials, unhampered by strategic aims. Indeed, in the essays the Darwinian idea of Blind Variation and Selective Retention was never discussed as an evolutionary account of creative career trajectories (see Simonton, 1997), but rather as an account of particular creative processes (see Johnson-Laird, 2002). The few students who mentioned the theory saw blind generation as akin to free, imaginative play, or “wild experimentation” (P2), which contrasts both with the idea of orderly processes and with strategic planning. The finding that compositional experience increased receptivity to such playful attitudes indicates a stark contrast to 20th-century's modernist notions of systematic pre-compositional planning (e.g., Stockhausen, 1964; Boulez, 1981; Xenakis, 1990), and to ideas of creativity as anxious struggle against predecessors (discussed with the students during the course: Straus, 1990).

The second research question was about whether students' argumentative strategies in the essays would vary between the theories and/or between the musical target activities. For addressing the question, nine classes of justificatory strategies were identified in the students' texts, subsequently observing to what extent these different lines of argument appeared in connection with the 10 theories. For two of the argumentative means, the distribution of participants applying the strategy in the 10 theories differed significantly from the distribution of participants discussing the theories. First, references to participants' own personal experiences were particularly often combined with the Four-Stage Model. This might be explained by introspective access to the characteristic incubation–illumination sequence of the theory: it may be almost too easy to introspectively apply the sequence to episodes of one's own creative experiences. Patterns of action that can relevantly be described by the model can be a part of the life-worlds of creative persons themselves, as also indicated by Katz and Gardner's (2012) use of the theory in accounting for composers' processes on the basis of interviews. Second, in comparison to other justificatory strategies, references to values were significantly emphasized in accounts of Divergent Thinking. In other words, arguments for Divergent Thinking simply tended to emphasize the special value or essential role of divergent ideas for musical creativity. It may be noted that in the written summary used for the study, the core idea of the theory might not seem much more than a value judgment in itself: “It has been argued that the more remote an idea is […], the more likely it is to be original and potentially creative” (Kozbelt et al., 2010, p. 29). Hence, many of the students' arguments in this category could be seen as simply affirming a definition of creativity as divergent thought and assuming that creativity is valuable.

A similar analysis regarding the uses of justification for the four musical target activities yielded one central finding: the argumentative strategy of problematization was particularly accentuated in the case of improvisation. In the introduction, we saw that while research in composition has frequently referred to general theories of creativity, such references have been all but nonexistent in some important anthologies of improvisation research. The argumentation analysis suggests that the participants of the present study may have felt similar problems in applying theories of creativity to improvisation. To be sure, a large share of the problematizations concerned the Four-Stage Model (e.g., claiming the notion of incubation to be inappropriate for improvising in the moment). In the creativity literature, such problems are well-known: Fischer and Amabile (2009), for instance, distinguish multistage “compositional creativity” from “improvisational creativity” in which the creative process “is one single step” (Fischer and Amabile, 2009, p. 16). In the ratings, however, not only the Four-Stage Model but also Problem Solving, Problem Finding, and Investment Theory were rated especially low for improvisation (see Figure 1). Consequently, the two strongest theoretical dimensions in the above principal component analysis, too, were de-emphasized for improvisation (see Figures 2A,B). It may have appeared somewhat contrived to the participants to interpret the continuous activity of improvisation as solving or finding discretely identified problems (see Mazzola and Cherlin, 2009). Of course, this does not rule out the possibility that other theoretical accounts might more successfully interpret improvisation in related terms—say, as an activity of solving problems of interactive coordination (Saint-Germier and Canonne, 2020; see also Sawyer, 2003).

The third research question asked which of the theories the students might see as particularly suitable for music. The highest mean ratings were received by Amabile's (1996) Componential Theory which was apparently deemed quite suitable for all of the target activities. In the essays where the students could freely choose their favorite theories, the three theories most often defended were the Four-Stage Model (47% of the participants), Divergent Thinking (43%), and Systems Theory (40%). Notice that while the first and last of these theories also align with the theoretical dimensions of Orderly Process and Socio-Cognitive System identified through the ratings, the theory of Divergent Thinking appeared in the ratings in two different dimensions, as either Strategic or Darwinian Divergence. Interestingly, most of the other theories loading on these two dimensions were only rarely chosen to be defended by the participants in their essays. It seems, then, that the students were unwilling to embrace explicitly strategic thinking (Investment Theory, Problem Finding), and perhaps even more unwilling to defend processes of Blind Variation/Selective Retention in order to account for the origin of divergent thought. Simply put, questions of creative intention vs. randomness rarely emerged as the main concern of the participants' arguments. Avoiding notions of strategic investment or defiance (see Sternberg, 2018) as well as non-strategic Darwinian thinking, the students more often chose to account for musical creativity as individual staged processes, as valuable divergence, or as complex systems that are either internal or external to the creative individual.

Finally, the fourth research question addressed the students' conceptions of theories and theory choice in general. In their essays, none of the students—despite reading Frith (2012) during the course—voiced anything like theory skepticism that would dismiss general theories of creativity across the board. (Given their oftentimes harsh criticism of individual theories, it does not seem likely that this was due to ingratiation with their teacher.) Perhaps less surprisingly, none of the students either proposed full-scale reorientation of the theoretical domain (theory revisionism). Instead, most of the students tended toward moderate forms of theory eclecticism, often choosing to argue for some combination of two or three theories. Many of them also explicitly argued that musical creativity cannot be accounted for just by a single theory. Accordingly, theories of creativity were treated not so much as mutually exclusive alternatives, but rather as spotlights illuminating the phenomenon of musical creativity from various angles. This eclectic approach is similar to the basic orientation of Componential Theory that indeed received the highest mean ratings in the study. It should be noted, though, that the concise theoretical summaries used in the present study may have supported an eclectic approach to theory choice and even favored some theories. In particular, whereas Amabile's (1996) full account of the Componential Theory also includes a model of creative response generation couched in information-processing terms, and even principles for predicting levels of creativity, the theoretical summary used in the present study was theoretically less precise, simply listing the three components of the theory (domain-relevant skills, creativity-relevant skills, task motivation). This might help explain the high ratings: the summary might simply have been accepted as a bazaar of useful-sounding requirements for creativity, leaving room for the students' own theoretical thinking to connect the dots. In the field of music education, a similar, informally eclectic approach is present in Webster's (1990) model of creative thinking in music that combines ideas related to product intentions, enabling skills and conditions, and a core consisting of movement, in Wallas's (1926) stages, between divergent and convergent thinking.

Notice that while many of the theories discussed in this study could be seen as relatively complex belief systems (at least in their original contexts), the basic insights of at least some of them might also well be expressed as simple definitions (e.g., “creativity is divergent thinking” or “creativity is problem solving”). The students' theory eclecticism might thus be taken to demonstrate that many combinations between such rudimentary definitions are not contradictory or meaningless, but rather allow multi-perspectival views to the phenomenon at hand. In this sense, the students' individual approaches to theories sometimes resembled more comprehensive scholarly definitions of creativity that cover various aspects such as aptitude, process, environment, and social recognition (e.g., Plucker et al., 2004). Interestingly, even researchers' multi-aspect theoretical models of creativity sometimes do not amount to much more than definitions: a case in point could be Webster's (1990) abovementioned model of creative thinking in music. To what extent theories of creativity in general tend to collapse to definitions would require another study. Here, I just want to note that a definitory approach to theorizing about creativity might sometimes just serve the role of specifying the topic of investigation. For instance, in saying that “creativity is problem solving,” we might not intend to be making an empirically falsifiable claim about some independently identifiable states of affairs (referred to by the term “creativity”). Instead, we might just be suggesting how to identify instances of creativity in the first place. Accordingly, the participants in the present research seemed to use the theories more often to identify a phenomenon of interest than to explain one. Thus, in recording a person's acknowledgment of a definitory theory (e.g., “creativity is problem solving”), we might not yet have covered much of her belief system concerning creativity. Such a belief system—an “implicit theory” if you will—would also include aspects of how she understands problem solving and its role for some phenomena of interest, how she constructs her own identity in relation to such activities, and so on. As seen in the introduction, personal belief systems about creativity should not be equated with summaries of beliefs on a group level, but they should also not be equated with the scholarly theories endorsed by the individual. Quite often, the latter might just serve to broach a topic.

What has been said above also reveals a lot about the students' criteria for theory choice. Recalling Kuhn's (1977) list of criteria for theory choice, we may first take up the important question regarding accuracy. In the essays, 85% of the students used generalized illustrations to justify their favored theories, while agreement with other observations or knowledge was not quite as common: 45% of the students appealed to personal experience, 38% to authorities, and 34% to particular examples. As these categories imply, “factual” support for the theories was mainly sought through examples—many of which were drawn from the students' own experiences. In science education, students' justifications may appear inappropriate if they are based on personal experiences (see e.g., Sampson and Clark, 2008), but in the arts this should not be as clear, as it would rule out many aesthetic arguments. In any case, the notion of accuracy at play here has less to do with explanatory adequacy than with just some sort of “fit” or “coverage”—finding examples that would fit a given theoretical description of creativity. In other words, theories were often treated simply as “ideas with evidence” (see Dagher et al., 2004). It may be difficult to draw a sharp distinction between accuracy in this loose sense and some loose criterion of fruitfulness. Apart from the suggested applications of the theories to music research (32%), students' generalized illustrations often included brief hypothetical examples of what someone could do musically in accordance with a given theory.

Other possible criteria for theory appraisal were applied less often. While the task instructions prompted the participants to address issues of theory scope—this was already implicit in asking for separate ratings for different target activities—only a few participants in their essays explicitly mentioned broad scope as an argument for a particular theory. Problems of narrow scope were frequently acknowledged when a given theory was deemed unfit for a certain target activity, but such problems were solved by eclectically turning to other theories. Accordingly, issues of consistency were mostly apparent as theory complementation: 53% of the participants commented on how theories might support one another in the task of accounting for musical creativity as a broader phenomenon. As shown by the example of theory reinforcement in the end of the results section, this was sometimes done with great ingenuity. By contrast, questions of the theories' internal consistency or their consistency with other beliefs were not discussed in the essays. Likewise, concerns for simplicity were hardly mentioned at all. These findings are thus in line with Furnham's (1988) conclusions in his study concerning lay theories: “Few lay people undertake a formal evaluation of theories, preferring a more pragmatic evaluation” (Furnham, 1988, p. 226). The students' thinking was driven by pragmatic concerns of finding fitting examples and fruitful contexts of application, but they largely ignored formal aspects that might affect theory choice—such as arguments for broad scope, simplicity, and consistency.

The study undertaken here has some obvious limitations, chief among which is perhaps the range of theories chosen to be addressed. In designing the study, I relied on what seemed one of the most balanced and wide-ranging chapter-length accounts of creativity theories available at the time (Kozbelt et al., 2010), but this selection has just scratched the surface (for a recent review, see Kaufman and Glăveanu, 2019). In the field of music, future work might especially need to pay more attention to theories with an eye on the embodied and socially distributed aspects of creative processes (see Linson and Clarke, 2017; van der Schyff et al., 2018; Schiavio et al., 2020). Another potentially problematic aspect, pointed out by two anonymous reviewers, is to what extent the results simply reflect the students' understanding or recall of course content. Quite obviously, some of the students may have studied the handbook chapter more carefully than others in the beginning of the course, thus “knowing” the theoretical context better than others who had to rely more on the short descriptions provided. Consequently, some of the theories may even have been misunderstood by some of the participants. While this is a genuine methodological issue, it also arguably reflects the situated character of the whole undertaking. In asking people to appraise theories of creativity, we are relying on the participants' individual points of view nurtured by their experiences and understandings regarding creativity, and it seems impossible to except the theories themselves from such “subjective” understandings. As any scholarly dispute about theories would suggest, theories themselves can be understood in different ways, and in the present context I have simply attempted to make such varying understandings more transparent by also engaging with the qualitative differences inherent in the participants' responses.

In closing, let us wrap up the challenges that the present research might raise to creativity research. Despite the lay character of their justifications, the participants were quite experienced musically, most of them reported rich creative activities, and they were all studying musicology or music education in the university, many at graduate level. In this sense, their views should arguably be taken more seriously than simply dismissing them as inaccurate if they do not align with research (cf. Introduction). Consider the case of improvisation. Jordanous and Keller (2012) have previously demonstrated that written accounts regarding improvisation might only emphasize a part of the concepts that characterize texts on creativity. The present results complement such findings by relating the problems of conceptualizing improvisation to theories of creativity. While some of the problems may be well-understood—e.g., the problems that the Four-Stage Model would have as a theory of improvisation—the study also reveals other aspects. The four theories that were least often chosen as favorite accounts of improvisation in students' essays—Blind Variation/Selective Retention, Investment Theory, Problem Finding, and Problem Solving—were also the least often defended theories overall. This suggests that improvisation may hold some keys to the intuitive acceptability of these theories. Of course, it remains open to the creativity theorist to hold that some such theories are not even intended to capture practitioners' beliefs about musical creativity.

Accordingly, such potential distance between well-received theories and musical practitioners' accounts of creativity should urge theorists to clarify on what basis their theories should be embraced. Or, in general, we may ask what theories of creativity are theories about, and in what sense they are theories. Supposing, for instance, that scientific theories should function as solutions to empirical problems (Laudan, 1977), the students' arguments analyzed in the present study would seem to fail to demonstrate a “scientific” use of the theories. Rather than as solutions to problems, theories were often used merely as descriptions that could fit some phenomena that the writer was familiar with. For the creativity theorist, possible responses might be either to show how her theory can be made to solve genuine problems, or to reject the suggested requirement for problem solving and explain what alternative functions the theory might serve. Similar points could be made regarding possible criteria for theory choice. In a sense, the pragmatic character of students' theory appraisals is not far from what was informally observed about music researchers' typical use of creativity theories in the introduction: in these applied contexts, explicit arguments regarding choice between theories of creativity are rarely put forward. For the creativity theorist, this poses challenges, one of which is not to propose yet a new theory of creativity without clearly articulating what its scope is supposed to be, and on what grounds it should be considered as rival to certain other theories.

At the outset, we saw that research on informal conceptions of creativity has been motivated by the idea that we should seek to align practitioners' views with research-based knowledge (Andiliou and Murphy, 2010). If so, the obvious step to take should be to actually engage students and practicing professionals with research on creativity. In the present study, I chose to do this with higher-education music students, but without assuming that the primary task was to “correct” them in their possible misconceptions. Instead, I have assumed that the students possess a wealth of first-hand experience in musical creativity and that their theory appraisals might thus tell us something important about the scope and nature of theories of creativity. If we are interested in fostering creativity in higher music education, we should arguably encourage students to engage with these theories with a similar sense of creative possibility that we expect to find in their music. Whatever philosophical conceptions we might hold regarding theory choice in research, as educators we probably should have no reason to argue against the students' pragmatic understandings of which ideas “work best” in relation to their own culturally specific and situationally changing creativities.

## Data Availability Statement

The datasets presented in this article are not readily available because the dataset includes qualitative case descriptions, with information that could reveal the identity of the participants. Requests to access the datasets should be directed to erkki.s.huovinen@gmail.com.

## Ethics Statement

Ethical review and approval was not required for the study on human participants in accordance with the local legislation and institutional requirements. The patients/participants provided their written informed consent to participate in this study. Written informed consent was obtained from the individual(s) for the publication of any potentially identifiable images or data included in this article.

## Author Contributions

The author confirms being the sole contributor of this work and has approved it for publication.

## Conflict of Interest

The author declares that the research was conducted in the absence of any commercial or financial relationships that could be construed as a potential conflict of interest.
