# Supplementary material for: Theories of Creativity in Music: Students' Theory Appraisal and Argumentation
Source: Front Psychol. 2021 Mar 25;12:612739. doi: 10.3389/fpsyg.2021.612739 (PMC8027343; doi:10.3389/fpsyg.2021.612739)
Supplement: Supplementary file 1 [file Data_Sheet_1.docx]

| Appendix 1. Theory descriptions as extracted from Kozbelt et al. (2010). | |
| --- | --- |
| **Title used** (with reference to the handbook section) | **Description**^1^ |
| **Developmental View**  (section *Developmental Theories*, p. 26) | “[T]hese theories imply a trajectory that starts with more subjective forms of creativity […] and develops into more tangible and mature forms of creative expression.” Some developmental theories have been “devised by examining the lives and family backgrounds of creative persons […]. These [have] suggested that particular developmental experiences [are] correlated with creativity. For instance, parents of creative children seem[…] to expose their children to diverse experiences and most [are] themselves in some ways creative.” |
| **Divergent Thinking**  (section *Psychometric Theories*, p. 29, and *Cognitive Theories*, p. 32) | “Divergent thinking occurs when ideas and associations move in varied directions, and as a result new and original ideas may be found […]. Convergent thinking, on the other hand, occurs when cognition is used to identify one correct or conventional answer. Divergent and convergent thinking can both be involved in creative efforts, which allows the generation of ideas that are both original and effective.” “It has been argued that the more remote an idea is (i.e., the farther from the starting point), the more likely it is to be original and potentially creative.” |
| **Investment Theory**  (section *Economic Theories*, p. 32) | Some researchers have “emphasized investments in creative behavior. Briefly stated, they [have] advocated the idea that creativity sometimes results when an individual buys low (i.e., invests in an idea that is currently unpopular) and then sells high (i.e., the idea gains respect).” |
| **Four-Stage Model**  (section *Stage and Componential Process Theories*, pp. 30–31) | According to the Four-Stage Model, a creative process “begins with a *preparation* stage where the individual gathers information and defines a problem. Next comes *incubation*, which involves taking some time away from a problem, at least consciously. If incubation is effective, a third stage occurs: insight, or what” has been “called *illumination*. At this point, a solution or idea suddenly makes itself known.” […] In the original version of this theory, “the final stage was *verification*. At that point, the individual tests the idea or applies the solution.” |
| **Componential Theory**  (section *Stage and Componential Process Theories*, p. 31) | Some researchers “have defined the creative process in terms of component mechanisms rather than stages […].” One of these componential models ”includes three facets: domain-relevant skills (e.g., knowledge about the domain, technical skills), creativity-relevant skills (e.g., appropriate cognitive style, knowledge of heuristics for generating novel ideas), and task motivation (e.g., attitudes toward specific tasks, perceptions of one’s motives).” |
| **Problem Solving**  (section *Theories Based on Problem Solving and Expertise*, pp. 33–34) | “Problem solving has usually been studied in puzzle-problems […], but its principles also apply to ill-defined problems […]. Such problems, like […] designing a house, have goals and operators that are not pre-specified and that admit multiple ’good-enough’ solutions, rather than one ’correct’ answer.” It has been argued “that ill-defined problems can often be broken into a set of well-defined problems, which can then be solved in familiar ways.” Hence, “the problem solving […] view regards creativity as an essentially rational phenomenon […], as amenable to meaningful strategic guidance and long-term learning.” |
| **Problem Finding**  (section *Problem-Finding Theories*, p. 34) | “The problem-finding view holds that the traditional problem-solving view is inadequate to explain how creators come to realize that a problem exists in the first place, and how they are motivated to proactively bring their subjective experience to understand the problem. In this view, heuristic search through a problem space simply does not apply to situations like making a painting, since there is no pre-specified set of alternatives to comprise the problem space. Problem finding is widely regarded as independent of problem solving, and it is mainly a theory of the creative process; it can also be seen as a theory of the creative person, assuming that something like the propensity for identifying interesting problems represents a stable personality variable.” |
| **Blind Variation/ Selective Retention**  (section *Evolutionary Theories*, pp. 35–36) | “The basis of [this] Darwinian model is a two-stage mental process, involving the blind generation and selective retention and elaboration of ideas […]. In this view, ideas are combined in some blind fashion, typically below the threshold of awareness; the most interesting combinations are then consciously elaborated into finished creative products […]. [B]ecause of the sheer complexity of the creative process, creators should have little control over guiding the process of their works; thus, it has been claimed that the creative process is replete with false starts and wild experiments […].” |
| **Seekers/Finders**  (section *Typological Theories*, pp. 37–38) | It has been argued “that there are two fundamental types of creators: aesthetically motivated experimentalists, or ‘seekers,’ and conceptual innovators, or ‘finders.’ The two types differ in how they approach the creative process, as well as in their career trajectories and the basis of their reputations. For seekers, the creative process is a frustrating struggle. Often eschewing preparatory work, they typically begin without a clear idea of their goals, proceed by trial and error, labor over their decisions, and have a difficult time declaring a work completed, using mainly perceptual criteria to do so. […] In contrast, finders frequently make detailed preparations and clearly know their goals at the outset. They thus typically work very efficiently and can easily decide when a project is finished.” |
| **Systems Theory**  (section *Systems Theories*, p. 39) | “Rather than regarding creativity as an intrinsic attribute of particular artifacts,” some researchers have “argued that creativity judgments emerge via three interacting components: 1) the domain, or body of knowledge that exists in a particular discipline at a particular time; 2) the individual, who acquires domain knowledge and produces variations on the existing knowledge; and 3) the field, comprised of other experts and members of the discipline, who decide which novelties produced by all of the individuals working in the discipline are worth preserving for the next generation. Each has a say in what counts as creative.” |
| ^1^ Square brackets indicate words or phrases added to the original wordings for clarity, or places where references or other words have been removed from the citations. Quotation marks and square brackets were removed for presentation to the participants. | |

| Appendix 2. Categories for argumentative content in the essays, with examples concerning the theory Divergent Thinking (participant numbers in parentheses). | | |
| --- | --- | --- |
| CLAIMS | Positive claim | “Among the presented theories, the one concerning divergent thinking best describes improvisation” (P15). |
|  | Negative claim | “However, divergent thinking is not suitable for understanding the performance of precomposed music” (P16). |
|  | Describing/highlighting theory | “The level of divergence in thought is principally measured by how ideas deviate from an individual’s own norms and from ordinary thinking. It is not before we assume some socially defined standard such as the tradition of Western art music that we can examine the novelty of ideas in a broader perspective” (P10). |
|  | Free generalization | “When there are both practical people and mad creators in a group, the end product, too, can be original, and still practically realizable” (P12). |
| JUSTIFICATIONS | Generalized illustration | “The distinction between divergent and convergent manages to express the compositional spiral, and such stages that can be discerned in composers’ work, both through sketches and by their own accounts” (P10). |
|  | Personal experience | “According to my own experience, this sort of thinking works, and yields good results in producing ideas for music in general, and in improvisation and composition in particular” (P16). |
|  | Particular example | “There are also composers like Schubert who could be very productive in an improvisatory manner. In this case, convergent thought works like an active filter, whereby the ideas come out already in a suitable format for Western art music” (P10). |
|  | Appeal to values | “As in composition, also in improvisation divergent thinking can be a double-edged sword: too ‘jazzy’ playing by a dance-band guitarist can lead to the audience’s disapproval. Still, many also find pleasure in the surprise and nonconformity of improvisation” (P4). |
|  | Appeal to authority | “While thinking about this I am struck by Nettl’s idea of building blocks which are used in a situation—perhaps even in a non-conscious manner” (P12). |
|  | Theoretical reasoning | “If the feeling of novelty is taken as one of the necessary aspects of creativity, divergent thinking could be seen as a downright requirement for creativity” (P14). |
|  | Theoretical complementation | “Thus the artist’s creative process begins in problem finding, proceeds through divergent thinking and action, and the end products (that may be many, in different phases of the divergent action) are subjected to the judgment of the field and the other experts who finally determine if the products are creative or not” (P37). |
|  | Application to research | “In the framework of this theory, it would be interesting to find out whether it could be useful in the treatment of mental health patients to help a schizophrenic who has good musical ideas to carry out these ideas in a therapeutic sense.” (P47) |
|  | Problematizing the theory | “The process of improvisation frequently requires a conscious, steadfast knowledge of the tradition, whereby an excessive personality in improvising may in some contexts be disadvantageous from the point of view of evaluating the final product. This can be seen, for instance, in the folk-musical improvisation of many countries, where the limits set by tradition preclude excessive originality. Thus, there is room for convergent thinking in improvisation” (P47). |
